# Clinical Characteristics Related to Central Lymph Node Metastasis in cN0 Papillary Thyroid Carcinoma: A Retrospective Study of 916 Patients

**DOI:** 10.1155/2014/385787

**Published:** 2014-08-21

**Authors:** Lie-Hao Jiang, Chao Chen, Zhuo Tan, Xiao-Xiao Lu, Si-Si Hu, Qing-Liang Wang, Xiu-Xiu Hou, Jun Cao, Ming-Hua Ge

**Affiliations:** ^1^Zhejiang Cancer Research Institute, Zhejiang Province Cancer Hospital, 38 Guangji Road, Gongshu District, Hangzhou 310022, China; ^2^Department of Head and Neck Surgery, Zhejiang Province Cancer Hospital, 38 Guangji Road, Gongshu District, Hangzhou 310022, China

## Abstract

*Background.* Papillary thyroid carcinoma (PTC) is a form of thyroid cancer with high risk of cervical lymph node metastasis. *Aim.* The aim of this study was to investigate the incidence and the predictive factors for occult ipsilateral central lymph node (CLN) metastasis in the patients with papillary thyroid carcinoma. *Methods.* A total of 916 PTC patients (1017 lesions) undergoing central lymph node dissection in our hospital from 2005 to 2011 were enrolled. The relationship between CLN metastasis and clinical factors such as gender, age, tumor size, tumor number, capsule invasion, and tumor location was analyzed. *Results.* Occult CLN metastasis was observed in 52.41% (533/1017) of PTC lesions, respectively. Multivariate analysis showed that age ≤ 35 years, tumor size > 1.5 cm, present capsule invasion/extracapsular invasion, and tumor located in upper/middle pole/whole lobe were risk factors of CLN metastasis. *Conclusions.* Tumor located in upper/middle pole/whole lobe, less than 35 years old, tumor size > 1.5 cm, and present capsule invasion/extracapsular invasion were risk factors of CLN metastasis. We recommend performing ipsilateral prophylactic CLN dissection in cN0 PTC patients.

## 1. Introduction

Papillary thyroid carcinoma (PTC), the most common form of thyroid gland carcinoma, accounts for more than 80% of thyroid carcinomas and 1% of all human malignant tumors. Despite the good prognosis, there are more than 50% PTC patients with the experience of cervical lymph node metastasis [1], which is associated with a higher rate of locoregional recurrence and distance metastasis [2]. Cervical lymph node was divided into seven levels according to the standard of classification proposed by the American Head and Neck Society (AHNS) and American Academy of Otolaryngology-Head and Neck Surgery (AAO-HNS) [3, 4], and CLN (Level VI) is the most common site for cervical lymph node metastasis in PTC patients.

Currently, it has been widely accepted that CLN dissection is necessary for the clinical lymph node positive (cN+) patients, while the need for prophylactic CLN dissection in clinical lymph node negative (cN0) patients remains controversial [5]. However, new evidence from a large scale nested case-control study suggested that patients with lymph node metastasis experienced a higher mortality, and the incomplete surgical excision was an important reason for the increased mortality in PTC patients of stage I [6]. Thus, in spite of the controversy on treatment, an increasing number of scholars propose to perform prophylactic ipsilateral CLN dissection on cN0 PTC patients.

The aim of this retrospective study was to evaluate the candidate predictive factors of CLN metastasis, in order to make a more appropriate selection of patients for prophylactic neck dissection.

## 2. Materials and Methods

### 2.1. Patients

A total of 916 patients who were first treated in the Department of Head and Neck Surgery, Zhejiang Cancer Hospital, between January 2005 and December 2011, were evaluated retrospectively. All the patients were pathologically diagnosed as PTC. Patients with other types of thyroid malignancy, with clinical positive lymph node, or with tumor in the isthmus were excluded. Patients with history of neck surgery for other diseases or radiation exposure were excluded. Among all patients, 815 patients were diagnosed with PTC in unilateral and 101 patients in bilateral; there were 186 males and 730 females with the male : female ratio of 1 : 3.92; the age of the patients ranged from 12 to 82 years with a median age of 43.80 years; the diameter of the tumors ranged from 0.1 cm to 6.0 cm with a median diameter of 1.1 cm.

### 2.2. Preoperative Ultrasonography and Tumor Location

Ultrasonography (US) was performed in all 916 patients to determine the lymph node status and tumor location. Tumor number was also decided by preoperative ultrasonoscopy and lesions were divided into solitary nodule group with only one nodule and multiple nodules group with more than one nodule in ultrasonoscopy. A total of 635 lesions with only one nodule, which was confirmed as PTC by paraffin section postoperatively, in ultrasonoscopy, were included in the groups of tumor location. Tumor location of lateral lobe was categorized as upper pole, middle pole, and lower pole according to the upper and lower level of isthmus. Tumors in upper pole were defined as located above the upper level of isthmus; below the lower level of isthmus was lower pole and the rest part of lateral lobe was middle pole. When the tumor covered one boundary, the tumor location was determined by the center of the tumor. When the tumor occupied almost whole lobe or covered two boundaries, it was classified as a new type—whole lobe.

### 2.3. Surgery

The patients with bilateral PTC underwent total thyroidectomy and bilateral CLN dissection, while total thyroidectomy or unilateral lobectomy plus isthmusectomy and ipsilateral CLN dissection were performed for unilateral PTC patients. Total thyroidectomy plus isthmusectomy might be considered when unilateral PTC patients met one or more of following conditions: tumor size > 4 cm, multifocal in one lobe, extrathyroid invasion, or distant metastasis, according to the guidelines of Chinese Thyroid Association. The CLN was level VI lymph nodes including the pretracheal and paratracheal nodes, precricoid (Delphian) node, and the perithyroidal nodes including the lymph nodes along the recurrent laryngeal nerves [3].

### 2.4. Grouping

One hundred and one patients with bilateral lesions were regarded as 202 independent lesions and there were a total of 1017 lesions included in the group. The patients who underwent CLN dissection were divided into different groups according to gender, age, tumor size, tumor number, tumor location, and capsule invasion (Table 1). Tumor number and tumor location were based on preoperative ultrasonoscopy and capsule invasion was based on intraoperative findings.

### 2.5. Statistics Analysis

Statistics analysis was performed using Statistical Package for Social Sciences (SPSS, Inc., Chicago, IL, USA). Univariate analysis was performed using chi-square criterion while multivariate analysis was performed using logistic regression analysis. A difference was considered statistically significant when *P* < 0.05.

## 3. Results

There were 53.71% (492/916) of patients and 52.41% (533/1017) of lesions confirmed with histologically positive central lymph node (CLN) metastasis. In the univariate analysis, CLN metastasis was significantly associated with gender, age, tumor size, capsule invasion, and tumor location (*P* < 0.01) and no significant correlation was found between tumor number and CLN metastasis (*P* > 0.05) (Table 2).

The rate of CLN metastasis decreased obviously with the increase of age in a certain range and there were significant difference in the rate of CLN metastasis between groups with age ≤ 25 years and 25 years < age ≤ 35 years, 25 years < age ≤ 35 years, and 35 years < age ≤ 45 years (*P* < 0.05), while no significant difference in the rate of CLN metastasis was found between the groups with age > 35 years (*P* > 0.05) (Table 3). Therefore, we regrouped the patients based on age ≤ 35 years and age > 35 years and found the CLN metastasis rate was significantly higher in group with age ≤ 35 years than age > 35 years (*P* < 0.01) (Table 2).

There were significant differences in the rate of CLN metastasis between the groups grouped based on the size of tumors and the rate of CLN metastasis increased obviously with the increase of tumor size in a certain range. There were significant differences in the rate of CLN metastasis between groups with Φ ≤ 0.5 cm and 0.5 cm ≤ Φ ≤ 1.0 cm, 0.5 cm ≤ Φ≤ 1.0 cm and 1.0 cm ≤ Φ ≤ 1.5 cm (*P* < 0.05), while no significant difference in the rate of CLN metastasis was found between the groups with 1.5 cm ≤ Φ ≤ 2.0 cm and Φ > 2.0 cm (*P* > 0.05) (Table 4). Accordingly, patients were redivided into group with Φ ≤ 1.5 cm and Φ > 1.5 cm and the CLN metastasis rate was significant higher in group with Φ > 1.5 cm than Φ ≤ 1.5 cm (*P* < 0.01) (Table 2).

It was also shown in the study that the location of the tumors in lobe was significantly related to CLN metastasis. The CLN metastasis rate of the group with tumor located in lower pole was significantly lower than other three groups (Table 5).

However, in the multivariate analysis, the rate of CLN metastasis was significantly higher in groups of age** ≤ **35 years (*P* < 0.05, odds ratio 3.14), tumor size > 1.5 cm (*P* < 0.05, odds ratio 3.69), present/extracapsular invasion (*P* < 0.05, odds ratio 1.76), and tumor located in upper/middle pole/whole lobe (*P* < 0.05, odds ratio 2.55) (Table 6).

## 4. Discussion

There is no debate on CLN dissection for cN+ PTC patients, while the need for prophylactic CLN dissection in cN0 PTC patients is still one of ongoing controversies. Traditionally, it has been accepted that cervical lymph node metastasis is associated with a higher rate of locoregional recurrence and distant metastasis but does not impair survival [2]. In addition, recent studies show that the morbidity rate of complications such as hypoparathyroidism or recurrent laryngeal nerve injury was significantly higher in the PTC patients who underwent lymph node dissection [7]. However, new evidence from a large scale nested case-control study suggested that patients with lymph node metastasis experienced a higher mortality and incomplete surgical excision is one of the reasons for the increased mortality of the patients with stage I differentiated thyroid carcinoma [6]. Besides, an increasing number of scholars support prophylactic CLN dissection for the reasons as follows: (1) recurrence of this compartment is hard to treat, (2) dissection can be done via the same incision as thyroidectomy, and (3) the incidence of metastasis never reaches zero even for low-risk cases.

The American Thyroid Association (ATA) Guidelines suggest that prophylactic CLN dissection can be considered, especially for patients with advanced primary tumors (T3 or T4), while prophylactic lateral lymph node (LLN) dissection is not recommended by ATA for the reasons of significant risks and the lack of impact on survival [8]. However, Ducoudray et al. [9] considered that prophylactic CLN and LLN dissection could modify PTC staging and simplify postoperative management for clinicians, so they recommend that selective prophylactic CLN and LLN dissection (ipsilateral to the main tumor) be performed in apparently node-negative PTC patients.

It is difficult to detect CLN metastases preoperatively because of the limitation of US for evaluating the central neck compartment. In addition, there has been no uniform evaluation criteria for cervical lymph node metastasis, but the existing thyroid carcinoma staging or prognosis rating systems, including systems of EORTC (European Organization for Research and Treatment of Cancer), MACIS (Metastases, Age, Completeness of Resection, Invasion, Size), and TNM (Tumor, Node, Metastasis) [10], may be of valuable reference. The evaluation factors of EORTC system include gender, age, histological type, capsule invasion, and distant metastasis. The evaluation factors of MACIS system include age, tumor size, extent of resection, local invasion, and distant metastasis. The evaluation factors of TNM system include age, tumor size, local invasion, regional lymph node metastasis, and distant metastasis. Therefore, referring to the above factors, the correlation between gender, age, tumor size, tumor number, capsule invasion, tumor location, and CLN metastasis was analyzed in our study.

It has been shown in many studies that women were more susceptible to papillary thyroid carcinoma. Glattre and Kravdal [11] considered that there was significant correlation between incidence of PTC and estrogen level in women, while male patients were more vulnerable to unhealthy lifestyles and harmful environmental factors, such as smoking and drinking. There were 186 males and 730 females with CLN dissection in our study with male : female ratio of 1 : 3.92, while the CLN positive rate of males was 66.13%, which, in the univariate analysis, was significantly higher than females (50.55%) (*P* < 0.01) (Table 2). However, in multivariate analysis, there was no significant difference in the rate of CLN metastasis between males and females (*P* > 0.05) (Table 6).

Although various staging systems listed age as one of the predictive factors of PTC prognosis, its role in CLN metastasis was controversial. Kutler et al. [12] analyzed 83 patients and considered that there is no significant difference in CLN metastasis between younger and older patients (42.6% and 38.9%, resp.; *P* = 0.82). In contrast, Vriens et al. [13] found that lymph node metastasis rate in adolescents and young adults group (15~39 years) was significantly higher than 40+ years group (31% and 22%, resp.; *P* = 0.008). Our results supported the latter. In the univariate analysis, the rate of CLN metastasis in group with age < 35 years was significantly higher than group with age ≥ 35 years (*P* < 0.01) (Table 2), which suggested that PTC patients less than 35 years old may be more susceptible to CLN metastasis.

Patients with tumor size greater than 1 cm were considered more susceptible to CLN metastasis [14]. Salter et al. [15] found that there were about 78% of CLN positive patients with the tumor size greater than 2 cm. It also has been reported that there was a statistically significant correlation between thyroid nodule size on ultrasound and cervical lymph node metastasis [16]. In our study, the rate of CLN metastasis increased obviously with the increase of tumor size in a certain range and the CLN metastasis rate was significantly higher in group with Φ > 1.5 cm than Φ ≤ 1.5 cm (*P* < 0.01) (Table 2). Besides, in the multivariate analysis, tumor size > 1.5 cm was one of the independent risk factors of CLN metastasis (*P* < 0.01, odds ratio 3.69) (Table 6).

An increasing number of scholars considered that tumor location in thyroid may be correlated to cervical lymph node metastasis. Delphian lymph node metastasis, a recognized indicator of further lymph node involvement in PTC, was considered be associated with tumor location in the isthmus or upper third of the thyroid [17]. Park et al. [18] suggested that skip lymph node metastasis occurred commonly with primary tumors of the upper pole and/or with tumors ≤ 1 cm in diameter. Besides, Liang et al. [19] found that tumor located in the middle/lower third of the lobe was independent predictor for central lymph node metastasis. However, there were still no uniform criteria for grouping based on tumor location. In our study, the lesions were divided into four groups according to the method mentioned above. In the univariate analysis, there was significant difference in the rate of CLN metastasis between those groups and the CLN metastasis rates in upper/middle pole/whole lobe groups were significantly higher than lower pole group (*P* < 0.01) (Table 5).

Capsule invasion has been reported in many studies as a risk factor of CLN metastasis. Vasileiadis et al. [20] believed that multifocality, bilaterality, size of tumor > 0.5 cm, and thyroid capsule invasion may have an increased risk of lymph node metastasis. The extrathyroid extension was presumed to increase the rate of CLN metastasis and have a negative effect on survival rate [21]. In this study, the CLN metastasis rate significantly increased with the increase of capsule invasion degree (*P* < 0.01) (Table 2). In the multivariate analysis, present/extracapsular invasion were independent risk factors of CLN metastasis (*P* < 0.01, odds ratio 1.76) (Table 6).

## 5. Conclusions

In summary, the rate of CLN metastasis was up to 52.41% in 1017 lesions, which indicated the generalization of occult CLN metastasis. Multivariate analysis in our study showed the existence of CLN metastasis was significantly related to tumor located in whole lobe or, upper and middle pole, less than 35 years old, tumor size > 1.5 cm, present capsule invasion, and extracapsular invasion. Therefore, we considered tumor located in whole lobe or, upper and middle pole, less than 35 years old, tumor size > 1.5 cm, present capsule invasion, and extracapsular invasion were risk factors of CLN metastasis. On this account, we recommend performing prophylactic CLN dissection in cN0 PTC patients.

## Figures and Tables

**Table 1:** Patient demographics and clinical characteristics.

Term	Case number	Percent
Gender		
Male	186	20.31%
Female	730	79.69%
Age (years)		
≤25	50	5.46%
25~35	159	17.36%
35~45	326	35.59%
45~55	247	26.97%
55~65	100	10.92%
>65	34	3.71%
Caspsule invasion		
Absent	645	63.42%
Present	171	16.81%
Extracapsular	201	19.77%
Tumor number		
Solitary	635	62.44%
Multiple	382	37.56%
Tumor size (cm)		
≤0.5	235	23.11%
0.5~1.0	352	34.61%
1.0~1.5	172	16.91%
1.5~2.0	93	9.14%
>2.0	165	16.22%
Tumor location		
Lower pole	159	25.04%
Middle pole	279	43.94%
Upper pole	163	25.67%
Whole lobe	34	5.35%

**Table 2:** Correlation between clinical factors and CLN metastasis: univariate analysis.

Term	Cervical lymph node metastasis				
	Negative	Positive	Case number	Positive rate	*P* value
Gender					0.000
Male	63	123	186	66.13%	
Female	361	369	730	50.55%	
Age (years)					0.000
≤25	5	45	50	90.00%	(0.000)∗
25~35	46	113	159	71.07%	
35~45	161	165	326	50.61%	
45~55	133	114	247	46.15%	
55~65	61	39	100	39.00%	
>65	18	16	34	47.06%	
Tumor size (cm)					0.000
≤0.5	181	54	235	22.98%	(0.000)∗∗
0.5~1.0	188	164	352	46.59%	
1.0~1.5	64	108	172	62.79%	
1.5~2.0	21	72	93	77.42%	
>2.0	30	135	165	81.82%	
Tumor number					
Solitary	307	328	635	51.65%	0.534
Multiple	177	205	382	53.66%	
Capsule invasion					
Absent	363	282	645	43.72%	0.000
Present	68	108	171	63.16%	
Extracapsular	53	148	201	73.63%	
Tumor location					0.000
Lower pole	106	53	159	33.33%	
Middle pole	127	152	279	54.48%	
Upper pole	68	95	163	58.28%	
Whole lobe	6	28	34	82.35%	

∗Age ≤ 35 years versus age > 35 years.

**Φ ≤ 1.5 cm versus Φ > 1.5 cm.

**Table 3:** The statistical analysis among groups grouped by age.

Age (years )	≤25	25~35	35~45	45~55	55~65
25~35	0.007				
35~45	0.000	0.000			
45~55	0.000	0.000	0.290		
55~65	0.000	0.000	0.042	0.224	
>65	0.000	0.000	0.693	0.921	0.409

**Table 4:** The statistical analysis among groups grouped by tumor size.

Tumor size (cm)	Φ ≤ 0.5	0.5 < Φ ≤ 1.0	1.0 < Φ ≤ 1.5	1.5 < Φ ≤ 2.0
0.5 < Φ ≤ 1.0	0.000			
1.0 < Φ ≤ 1.5	0.000	0.000		
1.5 < Φ ≤ 2.0	0.000	0.000	0.015	
Φ > 2.0	0.000	0.000	0.002	0.394

**Table 5:** The statistical analysis among groups grouped by tumor location.

Tumor location	Whole lobe	Upper pole	Middle pole
Upper pole	0.008		
Middle pole	0.002	0.437	
Lower pole	0.000	0.000	0.000

**Table 6:** Multivariate logistic regression for CLN metastasis.

Variables	*B*	S.E	Sig.	Exp(*B*)	95.0% CI Exp(*B*)	
					Lower	Upper
Gender (male versus female)	0.31	0.22	0.17	1.36	0.88	2.09
Age (≤35 years versus >35 years)	1.15	0.21	0.00	3.14	2.07	4.78
Tumor size (Φ > 1.5 cm versus Φ ≤ 1.5 cm)	1.31	0.22	0.00	3.69	2.41	5.67
Capsule invasion (present/extracapsular versus absent)	0.57	0.19	0.00	1.76	1.23	2.54
Tumor location (upper/middle/whole versus lower)	0.94	0.21	0.00	2.55	1.69	3.84
Constant	−1.52	0.21	0.00	0.22		
